# Ginseng-plus-Bai-Hu-Tang Combined with Western Medicine for the Treatment of Type 2 Diabetes Mellitus: A Systematic Review and Meta-Analysis

**DOI:** 10.1155/2022/9572384

**Published:** 2022-04-16

**Authors:** Min Zhou, Rong Yu, Xiu Liu, Xialin Lv, Qin Xiang

**Affiliations:** ^1^Hunan University of Traditional Chinese Medicine, Changsha 410208, China; ^2^Hunan Provincial Key Laboratory of Translational Research in Traditional Chinese Medicine Prescriptions and Zheng, Changsha 410208, China

## Abstract

**Objective:**

Type 2 diabetes mellitus (T2DM) is a chronic disease characterized by chronic hyperglycemia, which is also accompanied by changes in blood lipids and protein. According to research reports, Ginseng-plus-Bai-Hu-Tang (GBHT) has significant antihyperglycemic activity. Nevertheless, the evidence of effectiveness is not enough. In order to verify the effectiveness and safety of GBHT combined with conventional Western medicine (CWM) in the treatment of T2DM, we carried out this meta-analysis.

**Method:**

We collected 7 electronic databases from the inception to September 1, 2021; then, 12 studies were selected. The data analysis and methodological evaluation were conducted by the software RevMan 5.3.3 and Stata 12.0.

**Results:**

The meta-analysis revealed that when GBHT was adopted in combination with CWM, the effective rate (OR = 2.98, 95% CI = [2.01, 4.43], *P* < 0.00001), the FBG (MD = −0.86, 95% CI = [−1.06, −0.65], *P* < 0.00001), 2hBG (MD = −0.80, 95% CI = [−1.05, −0.55], *P* < 0.00001), and HbA1c (MD = −0.64, 95% CI = [−0.98, −0.30], *P* = 0.0002) of T2DM patients improved significantly compared with the control group. After GBHT combined with CWM treatment, HOME-RI (MD = −0.75, 95% CI = [−1.38, −0.12] *P* = 0.02) of T2DM patients was superior to CWM alone. In comparison, the benefit from FINS (MD = −1.42, 95% CI = [−4.46, −1.62], *P* = 0.36) was not apparent. In addition, none of the adverse events mentioned occurred, indicating that it is safe enough.

**Conclusion:**

GBHT combined with CWM is an effective and safe as adjunctive treatment for patients with T2DM. Nevertheless, due to the limitation of the quality of the included studies, additional high-quality researches are required to further confirm these results.

## 1. Introduction

In the past 30 years, the number of people with diabetes mellitus (DM) has quadrupled worldwide [1]. In the past ten years, the prevalence of DM in China has rapidly developed from a low prevalence rate (<3%) to a moderate prevalence rate (3–10%) [2]. It has been reported that there are 425 million diabetic patients in the world; additionally, by 2045, this number will grow to 629 million. According to the global burden of disease report, DM caused 1.37 million deaths in 2017 and has become the third largest noncommunicable disease threatening human health throughout the world, which is second only to cancer and cardiovascular diseases [3–5]. At present, in terms of the cost of diabetes treatment, the economic cost is high, with an amount reaching 20.86 billion, which accounts for 4.38% of the total medical cost; this situation has caused a considerable economic burden on individuals, families, and society [6, 7]. Increasing evidence has indicated that abnormalities in pancreatic islets, especially pancreatic *β*-cells, may be the central link in the onset of T2DM [8]. The main purpose of diabetes treatment is to prevent and treat various complications, as well as to delay the development of the disease and to improve the quality of life.

With the further exploration of multiple coactivation pathways for T2DM, it is unlikely that a single targeted therapy will work by itself. Due to the limitation of a single targeted therapy, more attention has been focused on combination therapy. When compared with a single CWM, the traditional Chinese medicine (TCM) prescription is a combination of multiple medicinal materials, which includes a variety of active ingredients and can be adopted for multiple simultaneous targeted treatments. When they are combined, they can provide perfect benefits and moderately function in a synergistic or antagonistic manner [9, 10]. Due to the advantages of low toxicity and their side effects, TCM is favoured by many patients for the treatment of T2DM.

Bai-Hu-Tang (BHT), which is composed of Gypsum fibrosum (Shi-Gao), rhizome of A. asphodeloides Bunge (Zhi-Mu), root of G. uralensis Fisch. (Gan-Cao) and seed of *O. sativa* L. (Jing-Mi) and is also a TCM that has been applied in China for more than 1800 years, was established by Zhongjing Zhang, who invented the six classic dialectical theory in the “Discussion of Cold Damage” and was referred to as a medical sage by later generations. GBHT is an enhanced formula of BHT that is prepared by adding Panax ginseng Meyer, which has significant antihyperglycemic activity [11–13]. GBHT is one of the commonly used prescriptions for T2DM in TCM, and it has the effects of clearing lung and stomach heat and for invigorating qi and yin; additionally, diabetic patients can be accompanied by a variety of symptoms, such as excessive thirst, polydrinking, and polyphagia. The ancient classical Chinese medicine theory holds that heat of the lung and stomach can cause excessive thirst, polydrinking, and polyphagia, which explains why GBHT can improve the symptoms of diabetic patients.

At present, a large number of clinical reports have been published on the effect of T2DM combined with CWM. Nevertheless, the evidence of its effect is still insufficient. Therefore, this study conducted systematic reviews of GBHT combined with CWM in the treatment of T2DM, with an aim of providing enough evidence and references for this scenario.

## 2. Methods

The protocol was registered on the International Platform of Registered Systematic Review and Meta-Analysis Protocols (INPLASY202220001), and it was conducted according to the preferred reporting items for systematic reviews and meta-analysis (PRISMA): The PRISMA Statement [14].

### 2.1. Search Strategy

We selected all clinical trials of GBHT combined with CWM for the treatment of T2DM. After extensive searches on various websites from their establishment to September 1, 2021, including EMBASE, PubMed, the China Science and Technology Journal Database (VIP), the Chinese Biomedical Literature Database (CBM), the Cochrane Library, the China National Knowledge Infrastructure (CNKI), and the Wanfang databases. Then, target literature were picked out. Manual searches would also be performed to track necessary references on related literature. The following were the search keywords and terms we used: “traditional Chinese medicine,” “Chinese medicine,” “ginseng-plus-Bai-Hu-Tang,” “Baihujiarenshentang,” “bai hu jia ren shen tang,” “Diabetes Mellitus,” “Diabetes Mellitus type 2,” “T2DM” OR “Diabetes,” “Xiaoke,” “Xiaodan,” “randomized controlled trial,” “Randomized,” ”clinical research,” and ”placebo.” See Supplemental File 1 for a full description of the search strategy (Supplemental File 1 search strategy).

### 2.2. Inclusion and Exclusion Criteria

#### 2.2.1. Inclusion Criteria

  (1) Study type: the included studies were RCTs studying GBHT combined with CWM for treating T2DM.
  (2) Type of patients: the patients were diagnosed with T2DM, regardless of race, nationality, gender, age, or course of disease.
  (3) Types of intervention: the experimental group was treated with GBHT combined with CWM, while the control group was treated with CWM alone (metformin, gliclazide, glipizide, rosiglitazone, etc.).
  (4) Types of outcome measures: the effective rate, fasting blood glucose (FBG), 2 hours postprandial blood glucose (2hBG), glycated hemoglobin (HbA1c), fasting insulin (FINS), and homeostasis model assessment of insulin resistance (HOME-RI). The effective rate was referred to “Guiding Principles for Clinical Research of New Chinese Medicines.” Standard of significantly effective: the significant improvement of symptoms and signs, including FBG, 2hBG, and HbA1c dropped ≥40%. Standard of effective: the obvious significant improvement of symptoms and signs, including FBG, 2hBG, and HbA1c decreased by ≥ 20%. Standard of invalidity: the clinical improvement of symptoms and signs did not reach the above standard.

#### 2.2.2. Exclusion Criteria

1. Non-RCTs and duplicate literature
2. Mechanism research, animal experiments, experience, and case reports
3. Studies with the incomplete data
4. Studies with unclear interventions, unclear description of efficacy evaluation criteria, or statistical errors

### 2.3. Data Abstraction

Two independent researchers (Min Zhou and Xiu Liu) conducted extensive screening and extracted target-related data for classification and integration. The extracted data included the first author, publication year, baseline characteristics, intervention, outcome indicators, and adverse events. In the process of screening, if we encountered difficulties that were difficult to resolve, we would discuss and decide in detail with the third researcher (Rong Yu).

### 2.4. Quality Assessment

Based on the Cochrane Systematic Review Manual RCT bias risk assessment tool, we completed the risk assessment of the included studies. The contents include the following: (1) random sequence generation, (2) allocation concealment, (3) blinding of participants and personnel, (4) blinding of outcome assessment, (5) incomplete outcome data, (6) selective reporting, and (7) other bias.

### 2.5. Statistical Analysis

We performed the meta-analyses with the help of RevMan 5.3.3 and Stata 12.0 software. Among them, odds ratio (OR) was used to evaluate binary variables. The mean difference (MD) and 95% CI were used when the continuous variables were of the same unit of measurement, and the standard mean difference (SMD) and 95% CI were adopted when the continuous variables were of different units of measurement. Heterogeneity was adopted to evaluate the effect; if *P* > 0.1 or *I*^2^ < 50%, the result was considered to be nonheterogeneity, and the fixed effects model was adopted. Otherwise, the random effects model was adopted, and the result indicated that the heterogeneity was significant, so the reasons for heterogeneity would be analyzed by performing subgroup analysis. Furthermore, sensitivity analysis would be conducted on each indicator to evaluate the stability, and the Egger test would be performed to test potential publication bias. *P* < 0.05 was considered statistically significant. GRADEpro software was used to access the strength of the evidence to make the results more credible. Finally, the TSA 0. 9. 5. 10 Beta software was used for sequential analysis of the test to explore the reliability of the analysis results.

## 3. Results

### 3.1. Selection of Study

**Figure 1:**
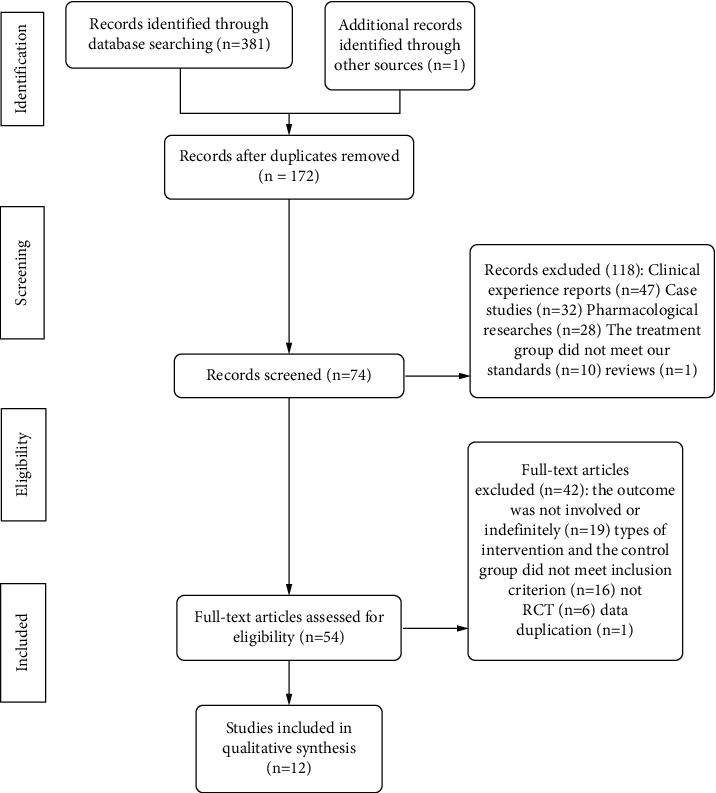
Flow of study selection.

A total of 381 potential literature were selected after extensive browsing and collection. 172 studies remained after we excluded 209 duplicates. And we excluded 118 literatures that did not meet the research objects by screening the title and abstract of the literature in detail. Immediately after that, we deleted 42 literature based on the inclusion criteria, and finally, we screened out 12 studies that met the inclusion criteria. The entire literature screening process was as shown in Figure 1.

### 3.2. Characteristics of the Eligible Studies

All the studies were carried out in China. The sample sizes of these trials ranged from 60 to 120. Treatment duration was from 1 to 6 months. Among the specific drugs for combination therapy, only 8 of which were treated with GBHT and metformin, 1 of which was performed with GBHT and gliclazide, 2 of which were conducted with glipizide, and 1 of which was conducted with rosiglitazone (Table 1).

### 3.3. Quality Assessment

**Figure 2:**
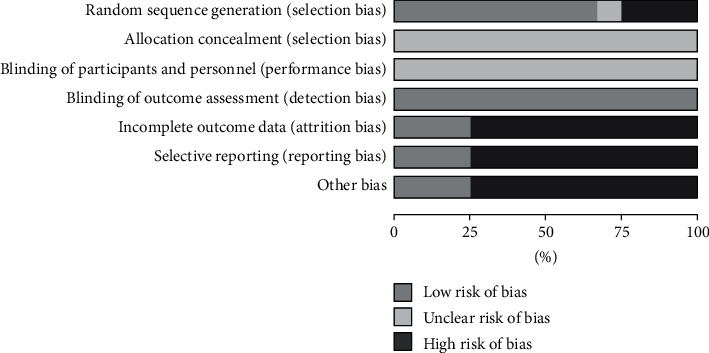
The risk of bias item presented as percentages across all included studies.

**Figure 3:**
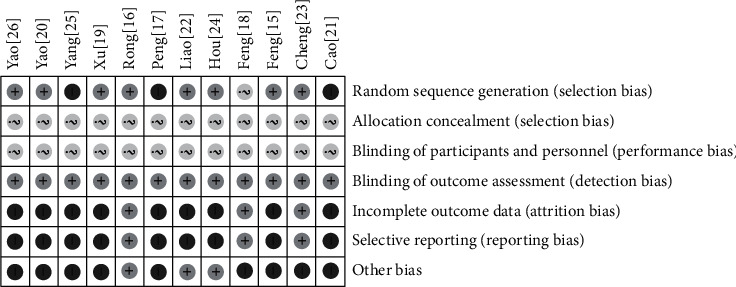
The risk of bias item for each included study.

The risk of bias (ROB) was implemented based on Cochrane criteria. Among them, three studies [17, 21, 25] were rated as “high risk” because they only mentioned randomness but did not mention specific random methods. Eight studies [15, 16, 19, 20, 22–24, 26] were rated as “low risk” due to the specific randomization methods reported. It was worth noting that one [23] of the studies mentioned the randomized controlled method in detail. There was one study [18] that did not mention any random method which was rated as “unclear.” None of the studies mentioned allocation concealment and whether to implement double blind; then it was rated as “unclear.” In terms of data integrity and selective reporting, three studies [16, 18, 23] reported whether there were adverse reactions and were rated as “low risk.” One study [23] described the dropout and withdrawal in detail. In terms of other risks, three studies [16, 22, 24] reported and implemented ethical review and passed the examination and approval, which were rated as low risk. Other studies were not involved, which were rated as high risk (Figures 2 and 3).

### 3.4. Evaluation of Meta-Analysis

#### 3.4.1. The Effective Rate

**Figure 4:**
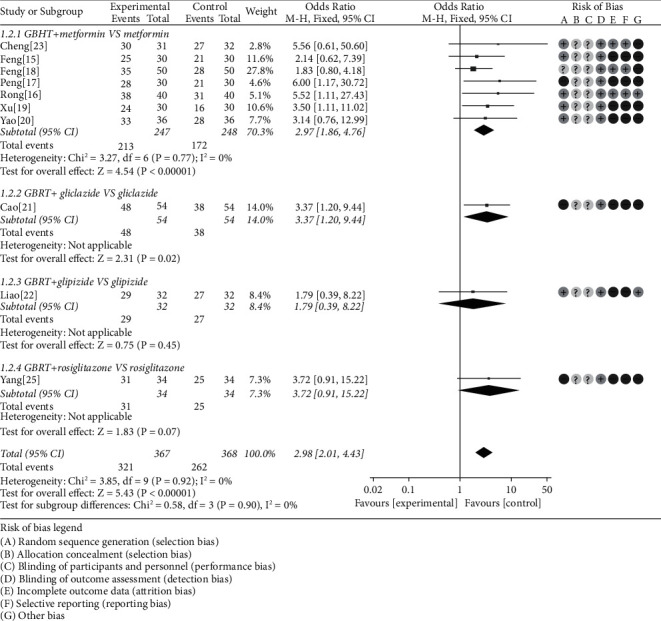
Meta-analysis on the effective rate of GBHT combined with CWM versus control group.

Odds ratio (OR) was adopted to analyze the efficiency according to the 10 included RCTs [15–23, 25]. Based on intuitive data analysis, the heterogeneity was not obvious (chi-square = 3.85, *P* = 0.92, *I*^2^ = 0%), and the fixed-effects model was used. Then, the analysis results suggested that the GBHT combined with CWM was superior to CWM alone (OR = 2.98, 95% CI = [2.01, 4.43], *P* < 0.00001). Further subgroup analysis showed that there was statistical significance of GBHT combined with metformin compared with metformin alone (chi-square = 3.27, *I*^2^ = 0%, OR = 2.97, 95% CI = [1.86, 4.76], *P* < 0.00001) and gliclazide (*P* = 0.02). In comparison, there was no statistical significance of glipizide (*P* = 0.45) and rosiglitazone (*P* = 0.07) (Figure 4).

#### 3.4.2. FBG

**Figure 5:**
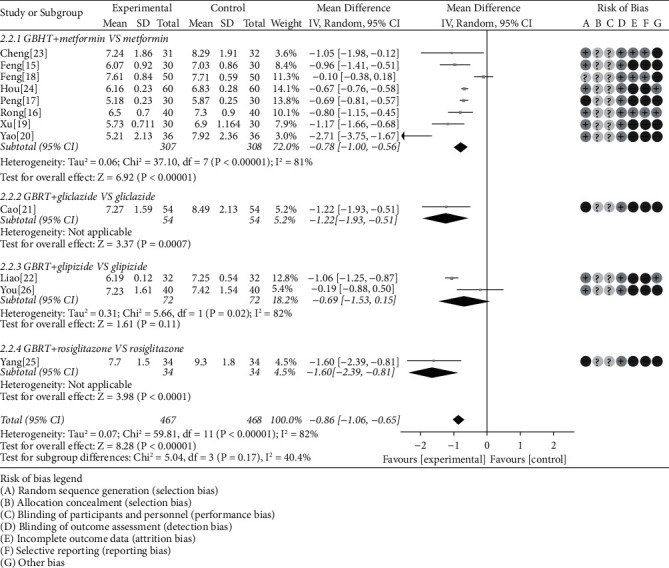
Meta-analysis on the FBG of GBHT combined with CWM versus control group.

The mean difference (MD) was used for the FBG based on the 12 studies [15–23, 26]. Data analysis results showed that there was obvious heterogeneity (chi-square = 59.81, *P* < 0.00001, *I*^2^ = 82%); then, we adopted the random-effects model. According to the data analysis, results illuminated that the GBHT combined with CWM was superior to CWM alone (MD = −0.86, 95% CI = [−1.06, −0.65], *P* < 0.00001). Further subgroup analysis indicated that there was statistical significance of GBHT combined with metformin compared with metformin alone (chi-square = 37.10, *I*^2^ = 81%, MD = −0.78, 95% CI = [−1.00, −0.56], *P* < 0.00001), gliclazide (*P* = 0.0007), and rosiglitazone (*P* < 0.0001). By contrast, the data analysis results showed no statistical significance in glipizide (*P* = 0.11) (Figure 5).

#### 3.4.3. 2hBG

**Figure 6:**
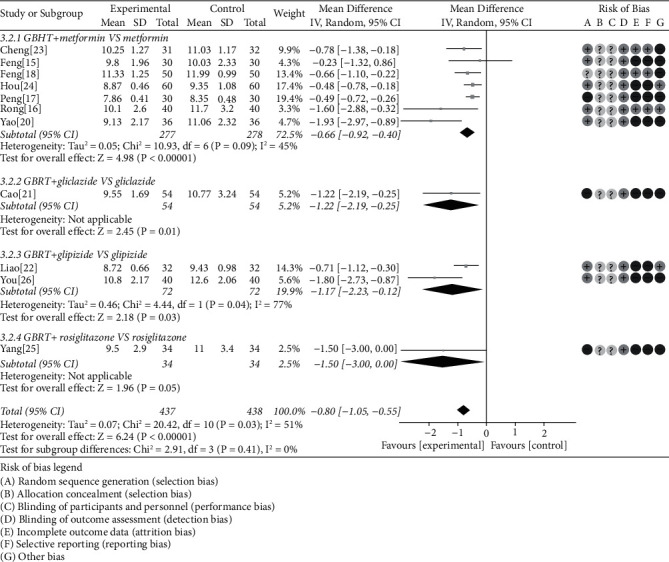
Meta-analysis on the 2hBG of GBHT combined with CWM versus control group.

11 studies were included to perform 2hBG [15–18, 20–25]. There was obvious heterogeneity (chi-square = 20.42, *P* = 0.03, *I*^2^ = 51%), and the random-effects model showed that the GBHT combined CWM was superior than CWM alone (MD = −0.80, 95% CI = [−1.05, −0.55], *P* < 0.00001). Data extracted suggested that there was statistical significance of GBHT combined with metformin compared with metformin alone (chi-square = 10.93, *I*^2^ = 45%, MD = −0.66, 95% CI = [−0.92, −0.40], *P* < 0.00001), gliclazide (*P* = 0.01), and glipizide (*P* = 0.03). On the contrary, analysis result displayed no statistical significance in rosiglitazone (*P* = 0.05) (Figure 6).

#### 3.4.4. HbA1c

**Figure 7:**
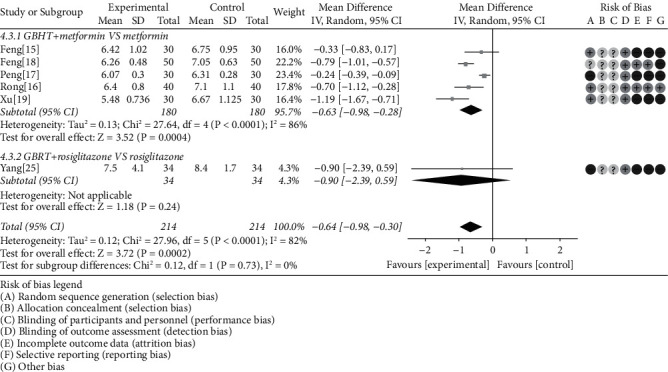
Meta-analysis on the HbA1c of GBHT combined with CWM versus control group.

6 literatures [15–19, 25] reported the change of HbA1c. Our pool findings revealed that there was apparent heterogeneity among the RCTS (chi-square = 27.96, *P* < 0.0001, *I*^2^ = 82%), and the random-effects model showed that the GBHT combined with CWM was superior to CWM alone (MD = −0.64, 95% CI = [−0.98, −0.30], *P* = 0.0002). The pooled subgroup analysis clarified that GBHT combined with metformin was statistically significant compared to metformin alone (chi-square = 27.64, *I*^2^ = 86%, MD = −0.63, 95% CI = [−0.98, −0.28], *P* = 0.0004). Nevertheless, it was of no statistical significance in rosiglitazone (*P* = 0.24) (Figure 7).

#### 3.4.5. FINS

**Figure 8:**
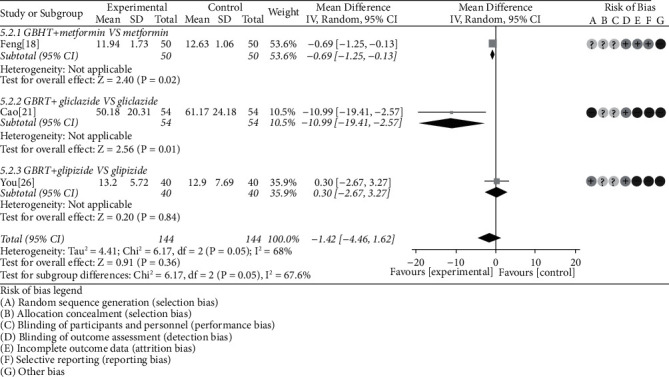
Meta-analysis on the FINS of GBHT combined with CWM versus control group.

3 studies [18, 21, 26] were provided on the change of FINS. According to the intuitive data, there was obvious heterogeneity (chi-square = 6.17, *P* = 0.05, *I*^2^ = 68%), and the random-effects model suggested that the GBHT combined with CWM was of no advantage compared with CWM alone (MD = −1.42, 95% CI = [−4.46, −1.62], *P* = 0.36). Detailed subgroup analysis showed that GBHT combined with metformin was statistically significant compared with metformin alone (*P* = 0.02) and gliclazide (*P* = 0.01). On the contrary, there was no statistical significance in glipizide (*P* = 0.84) (Figure 8).

#### 3.4.6. HOME-RI

**Figure 9:**
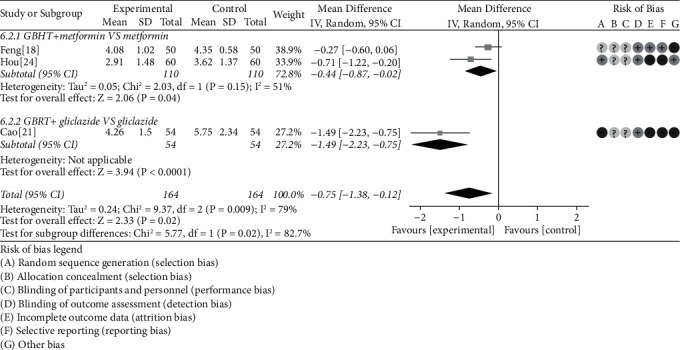
Meta-analysis on the HOME-RI of GBHT combined with CWM versus control group.

There were 3 studies [18, 21, 24] conducted on the analysis of HOME-RI. According to the visual data, there is obvious heterogeneity between these RCTS (chi-square = 9.37, *P* = 0.009, *I*^2^ = 79%); then, the random-effects model was adopted. Data analysis results showed that GBHT combined with CWM was statistically significant compared with CWM alone (MD = −0.75, 95% CI = [−1.38, −0.12], *P* = 0.02). Further subgroup analysis showed that GBHT combined with metformin is statistically significant compared with metformin alone (chi-square = 2.03, *I*^2^ = 51%, MD = −0.44, 95% CI = [−0.87, −0.02], *P* = 0.04) and gliclazide (*P* < 0.0001) (Figure 9).

#### 3.4.7. Sensitivity Analysis and Publication Bias

In order to explore the stability of meta-analysis, we performed sensitivity analysis to ensure that the results were not caused by one or two studies based on the effective rate, FBG, 2hBG, HbA1c, FINS, and HOME-RI. In the end, the sensitivity analysis results showed that the results were relatively stable. See Supplemental File 2 for all sensitivity analysis of the each outcome (Supplemental File 2 sensitivity analysis of Supplementary Figures 1–6).

In addition, we carried out Egger's test to detect the publication bias due to high heterogeneity and the test results suggested that it was of no significant difference in 2hBG, HbA1c, FINS, and HOME-RI (*P* > 0.05). Nevertheless, there was statistically significant in the FBG (*P* = 0.006 < 0.05), suggesting potential publication bias was identified. Due to the regional differences in TCM culture, all literature studies are carried out and completed in China, and the reported research results are all positive. Therefore, publication bias may be related to region, race, and unpublished negative results (Table 2).

#### 3.4.8. Outcome Indicators' Evidence Quality Rating

We evaluated the quality of evidence by the GRADEpro software, and the results presented that the reliability was low (Table 3).

#### 3.4.9. Test Sequential Analysis

**Figure 10:**
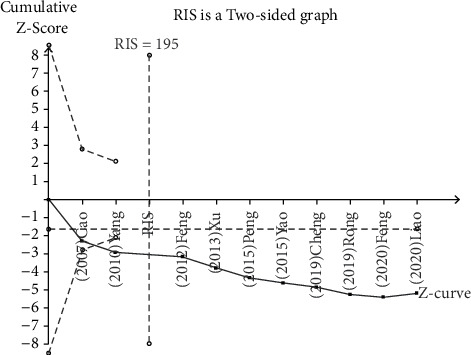
Test sequential analysis on the effective rate of GBHT combined with CWM versus control group.

Test sequential analysis (TSA) analysis was performed on the efficacy of GBHT combined with CWM in the treatment of T2DM. The sample size was adopted as the expected value (RIS). Based on the type I error probability (*α* = 0.05), the type II error probability (*β* = 0.2) and the RIS were estimated to be 195. The results shown that the cumulative *Z*-value (*Z* curve) intersected the expected information value in the second study, which has demonstrated that the results obtained by meta-analysis could be adopted as a definite conclusion. This result also revealed that GBHT combined with CWM was better than CWM alone for T2DM, which was reliable. Nevertheless, considering that this systematic review still has certain limitations, more high-quality RCTs are needed to further verify it (Figure 10).

## 4. Discussion

In TCM, diabetes is known as “Xiaoke,” and syndrome differentiation of Sanxiao theory is the most common method [27]. In the “Guidelines for the Prevention and Treatment of Diabetes in TCM” issued by the Chinese Society of TCM in 2011 [28], the current stage of diabetes was divided into the following types: mutual syndrome of phlegm and heat, syndrome of consumption of fluid due to intense heat, and qi-yin deficiency syndrome. From the perspective of TCM theory and practical experience, GBHT is often used to treat T2DM with lung and stomach heat syndrome and qi deficiency syndrome. Xichun Zhang, who is one of the famous Chinese medicine doctors in ancient times, demonstrated that the pancreas was the accessory organ of the spleen in the “Medical Integrative Chinese and Western Records” [29]. Therefore, the onset of diabetes in TCM occurs in the spleen, which is consistent with the theory that the key mechanism to T2DM in Western medicine is insulin resistance and islet B cell dysfunction. Theoretical research on the pathogenesis of diabetes in TCM is essentially consistent with the pathogenesis of diabetes in modern medicine. In addition, TCM has a long history of theoretical research and clinical prescriptions and has accumulated rich experience in clinical diagnosis and treatment, which is an incomparable advantage of TCM compared with modern medicine. With the advancement of modern medicine, an increasing number of pharmacological studies have gradually demonstrated the rationality of prescription and TCM compatibility treatment.

Botanicals exhibit antiobesity, blood fat-lowering, and antidiabetic effects and have been widely adopted in history. GBHT is a common herbal treatment that has been widely used to relieve the symptoms of thirst in patients with T2DM [11]. Laboratory research has shown that the combined effects of the five drugs of GBHT can effectively reduce the fasting blood sugar levels of diabetic rats. With the exception of japonica rice, all of the extracts of the other four drugs have been demonstrated to reduce blood sugar levels [30]. In addition, related studies have shown that GBHT can significantly reduce blood sugar and dry IFN-*γ* levels in diabetic young rats, as well as increase IL-4 levels, improve the pathological structure of the pancreas, and repair islet immune damage in diabetic young rats [31]. Moreover, experimental research has proven that GBHT effectively reduces blood sugar and blood lipid levels in diabetic rats and has a significant effect on the improvement of the insulin sensitivity index [32]. Simultaneously, GBHT can not only increase the weight of the rat spleen but can also improve the ratio of fasting C-peptide/blood sugar in rats, thus indicating that GBHT enhances immune function [33]. Related experimental studies have confirmed that GBHT can reduce blood glucose and glycosylated hemoglobin levels in rats and could significantly increase the activity of SOD in serum and decrease the content of MDA, suggesting that GBHT can protect pancreatic islet B cells by improving antioxidant capacity [34].

GBHT can not only regulate the glucose and lipid metabolism of T2DM rats but also improve diabetes-related complications. Experimental research has shown that the active component of GBHT can inhibit endothelial cell damage, enhance the function of vascular endothelial cells, and improve vasodilation and contraction, thereby eliminating vascular complications [35]. In addition, GBHT can upregulate the expression of glucose transporter 4 in the myocardium of diabetic rats induced by streptozotocin and can prevent the occurrence of diabetic cardiomyopathy [36, 37]. Moreover, GBHT also has the ability to regulate the TLR 4/NF-*κ*B signalling pathway, which improves intestinal barrier function and reduces intestinal inflammation, thereby improving insulin resistance and lowering blood sugar levels [38].

In our current study, we pooled the data from 12 studies involving 735 patients. Our pooled analysis revealed that when GBHT was adopted in combination with CWM, the FBG, 2hBG, and HbA1c levels of T2DM patients were significantly improved. After treatment with GBHT combined with CWM, the HOME-RI of T2DM patients was substantially enhanced. However, the improvement on FINS was not observed. In this meta-analysis, none of the adverse events mentioned occurred, indicating that it is safe enough. In addition, the GRADE analysis results showed that the reliability of the outcome indicators was mostly moderate and low. Therefore, we can carefully recommend GBHT as being an adjuvant treatment for T2DM.

However, this study had several limitations. First, the allocation concealment and blinding method were not described in all of the studies, and the reliability of the clinical research data collected in this case has yet to be verified. Second, some studies considered exercise and diet control as basic research, regardless of whether the master exercise was Tai Chi or aerobic or how long the exercise took to perform, which was not explained in detail. It is worth noting that some studies have shown that aerobic exercise can ameliorate the levels of visceral fat area and HbA1c in middle-aged and elderly obese patients with T2DM. This will also be a factor that affects publication bias. Third, TCM treatment of diseases is based on “syndrome,” and syndrome differentiation is the core link of TCM intervention. However, in clinical research, researchers often have a tendency to apply a certain drug to the treatment of T2DM, thus resulting in the weakening or even absence of syndrome differentiation and treatment. Moreover, the efficacy of T2DM intervention is mostly reflected in the longer time period after the intervention, making the evaluation of long-term efficacy particularly essential. However, there is a general lack of follow-up observations of the long-term efficacy of patients in clinical studies, and it is mostly limited to the short-term time period after drug intervention. Finally, there is still a lack of multicentre, large sample, prospective randomized controlled trials in clinical research, which weakens the reliability and persuasiveness of experimental data. Therefore, in future clinical research, further multicentre, large-sample, prospective studies should be performed to better explore the effect of TCM.

## 5. Conclusions

The results of our current analysis indicates that GBHT combined with CWM is more effective as adjunctive treatment for patients with T2DM. However, there are still some defects in this study, such as the small number of included literature, low methodological quality, and the existence of heterogeneity and publication bias. Therefore, clinical studies with high methodological quality and large sample size are still needed in the future to further verify the validity of GBHT combined with CWM for T2DM.

## Figures and Tables

**Table 1:** Characteristics of included studies.

Studies (first author, year)	Location	Sample size (male/female)	Age (mean years)	Interventions	Course of treatment (months)	Outcomes
Feng et al. [15], 2020	Jiangsu, China	E: 14/16 C: 12/18	E: 59.54 ± 5.39 C: 58.85 ± 6.15	E:GBHT + C C:metformin tid	6	①②③④
Rong [16], 2019	Guangdong, China	E: 15/10 C: 14/10	E: 56.4 ± 5.1 C: 55.9 ± 4.8	E: GBHT + C C: metformin tid	2	①②③④
Peng et al. [17], 2015	Guangdong, China	E: 13/17 C: 16/14	E: 49.8 ± 7.48 C: 50.5 ± 7.73	E: GBHT + C C: metformin tid	3	①②③④
Feng [18], 2012	Shandong, China	E: 16/14 C: 17/13	E:55.74 ± 7.44 C:56.33 ± 6.42	E: GBHT + C C: metformin bid	3	①②③④⑤⑥
Xu [19], 2013	Shanxi, China	E:17/13 C:18/12	E: 58.5 ± 8.21 C: 58.94 ± 8.79	E: GBHT + C C: metformin tid	6	①②④
Yao and Cheng [20], 2015	Zhejiang, China	33/39	48.64	E: GBHT + C C: metformin tid	1.5	①②③
Cao [21], 2007	Hunan, China	E: 26/28 C: 26/28	E: 56.7 ± 10.3 C: 54.5 ± 9.9	E: GBHT + C C: gliclazide bid	2	①②③⑤⑥
Liao e al. [22], 2020	Guangdong, China	E: 17/15 C: 13/19	E: 48.84 ± 5.07 C: 48.91 ± 5.30	E: GBHT + C C: glipizide tid	1	①②③
Cheng [23], 2019	Chengdu, China	E: 15/16 C: 17/15	E: 52.48 ± 13.71 C: 54.59 ± 4.70	E: GBHT + C C: metformin tid	3	①②③
Hou [24], 2017	Hubei, China	E: 37/23 C: 36/24	E: 47.59 ± 5.48 C: 48.65 ± 6.72	E: GBHT + C C: metformin tid	2	③⑥
Yang [25], 2010	Guangdong, China	E: 18/16 C: 20/14	47 ± 4	E: GBHT + C C: rosiglitazone tid	3	①②③④
You et al. [26], 2009	Hebei, China	E: 23/17 C: 27/13	E:57 ± 12.5 C:54 ± 14	E: GBHT + C C: glipizide tid	1	②③⑤

E: experimental group, C: control group; ①: the effective rate, ②: FBG, ③: 2hBG, ④: HbA1c, ⑤: FINS, and ⑥: HOME-RI.

**Table 2:** Summary of sensitivity analysis and publication bias of parameters.

	OR/MD fluctuation	95% CI fluctuation	Publication bias (*P* value)
The effective rate	0.09	(0.83, 1.00)	0.884
FBG	−1.02	(−1.17, −0.88)	0.006
2hBG	−0.62	(−0.80, −0.49)	0.334
HbA1c	−0.81	(−1.01, −0.61)	0.805
FINS	−0.33	(−0.60, −0.10)	0.165
HOME-RI	−0.52	(−0.75, −0.30)	0.987

Explanation: *P* < 0.05 indicates that a publication bias exists.

**Table 3:** Statement of evidence quality of GBHT combined with CWM in the treatment of T2DM.

GBHT plus CWM for T2DM						

Patient or population: [Patients with T2DM]. Setting:all eligible patients with intervention therapy Intervention: [GBHT + CWM,CWM]						

Outcomes	Illustrative comparative risks ^*∗*^ (95% CI)		Relative effect (95% CI)	No of participants (studies)	Quality of the evidence (GRADE)	Comments
	Assumed risk	Corresponding risk				
	Control	GBHT + CWM				

The effective rate	Study population		OR 2.98 (2.01 to 4.43)	595 (8 studies)	⊕⊕⊕Ο moderate^a^	
	701 per 1000	863 per 1000 (800 to 933)				
	Moderate					
	702 per 1000	863 per 1000 (800 to 934)				

FBG	See comment	See comment	The mean was MD 0.86 lower (1.06 to 0.65 lower)	935 (12 RCTS)	⊕ΟΟΟ very low^abc^	

2hBG	See comment	See comment	The mean was MD 0.80 lower (1.05 to 0.55 lower)	875 11 RCTS)	⊕⊕⊕Ο moderate^ab^	

HbA1c	See comment	See comment	The mean was MD 0.64 lower (0.98 to 0.30 lower)	428 (6 RCTS)	⊕⊕ΟΟ low^ab^	

FINS	See comment	See comment	The mean was MD 1.42 lower (4.46 to 1.62 higher)	288 (3 RCTS)	⊕⊕ΟΟ low^ab^	

HOME-RI	See comment	See comment	The mean was MD 0.75 lower (1.38 to 0.12 lower)	328 (3 RCTS)	⊕⊕ΟΟ low^ab^	

^*∗*^The basis for the assumed risk (e.g. the median control group risk across studies) is provided in footnotes. The corresponding risk (and its 95% confidence interval) is based on the assumed risk in the comparison group and the relative effect of the intervention (and its 95% CI). CI: Confidence interval; OR: Odds ratio; GRADE Working Group grades of evidence High quality: Further research is very unlikely to change our confidence in the estimate of effect. Moderate quality: Further research is likely to have an important impact on our confidence in the estimate of effect and may change the estimate. Low quality: Further research is very likely to have an important impact on our confidence in the estimate of effect and is likely to change the estimate. Very low quality: We are very uncertain about the estimate. Explanations: a. No blinding. b. High heterogeneity. c. *P* < 0.05 in Egger's test.

## Supplementary Materials

Supplementary Materials
*Supplemental File 1*. Search strategy. *Supplemental File 2*. Sensitivity analysis of supplementary Figures 1–6.
