# Supplementary material for: Ginseng-plus-Bai-Hu-Tang Combined with Western Medicine for the Treatment of Type 2 Diabetes Mellitus: A Systematic Review and Meta-Analysis
Source: Evid Based Complement Alternat Med. 2022 Apr 16;2022:9572384. doi: 10.1155/2022/9572384 (PMC9034934; doi:10.1155/2022/9572384)
Supplement: Supplementary Materials — Supplemental File 1. Search strategy. Supplemental File 2. Sensitivity analysis of supplementary Figures 1–6. [file 9572384.f1.zip › 9572384.f1/Supplemental File 1 search strategy.pdf]

Supplement 1: search strategy.

No.	Searches
#1	Diabetes Mellitus type 2 [MeSH]
#2	Diabetes Mellitus [MeSH]
#3	Diabetes [MeSH]
#4	Xiaodan [title/abstract]
#5	Xiaoke [title/abstract]
#6	#1 OR #2 OR #3 OR #4 OR #5
#7	Baihujiarenshentang [MeSH]
#8	ginseng-plus-Bai-Hu-Tang [MeSH]
#9	bai hu jia ren shen tang [MeSH]
#10	traditional Chinese medicine [title/abstract]
#11	Chinese medicine [title/abstract]
#12	#7 OR #8 OR #9 OR #10 OR #11
#13	randomized controlled trial [publication type]
#14	Randomized [title/abstract]
#15	clinical research [title/abstract]
#16	Placebo [title/abstract]
#17	#13 OR #14 OR #15 OR #16
#18	#6 AND #12 AND #17
