# Supplementary material for: Ginseng-plus-Bai-Hu-Tang Combined with Western Medicine for the Treatment of Type 2 Diabetes Mellitus: A Systematic Review and Meta-Analysis
Source: Evid Based Complement Alternat Med. 2022 Apr 16;2022:9572384. doi: 10.1155/2022/9572384 (PMC9034934; doi:10.1155/2022/9572384)
Supplement: Supplementary Materials — Supplemental File 1. Search strategy. Supplemental File 2. Sensitivity analysis of supplementary Figures 1–6. [file 9572384.f1.zip › 9572384.f1/Supplemental File 2 sensitivity analysis.docx]

Supplement 2: sensitivity analysis


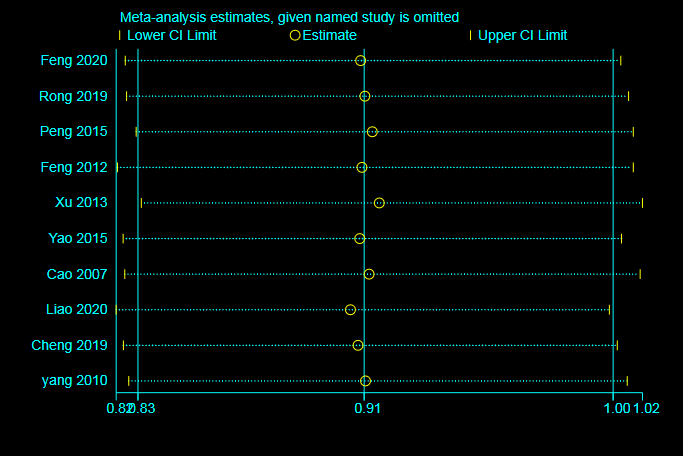


Supplementary figure 1: The sensitivity analysis of the effective rate.


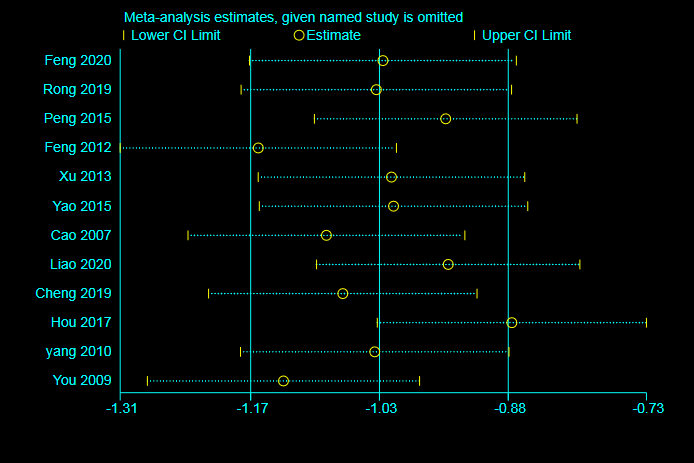


Supplementary figure 2: The sensitivity analysis of FBG.


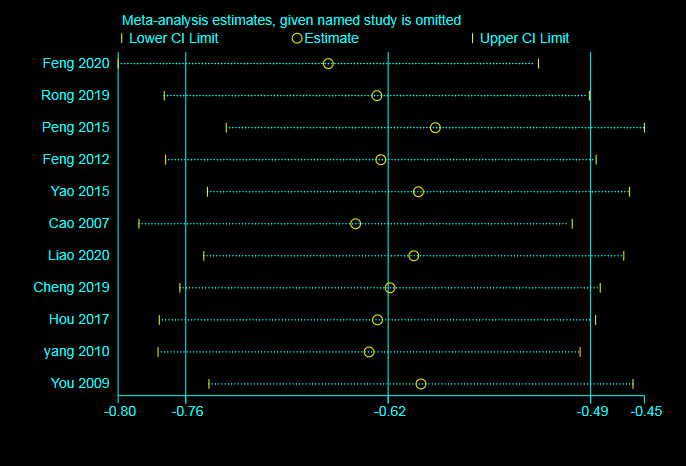


Supplementary figure 3: The sensitivity analysis of 2HBG.


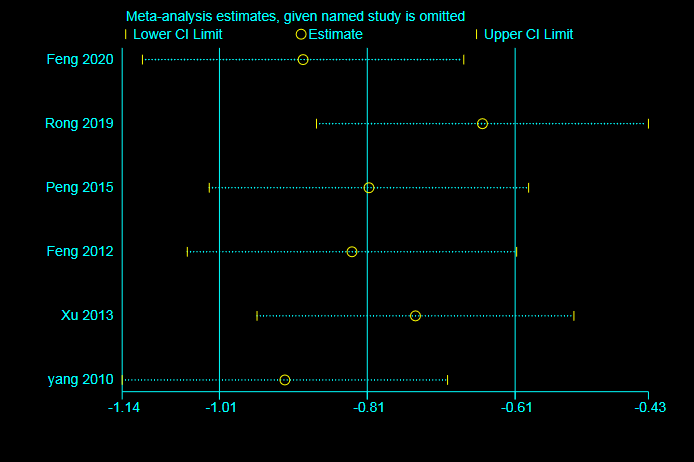


Supplementary figure 4: The sensitivity analysis of HbA1c.


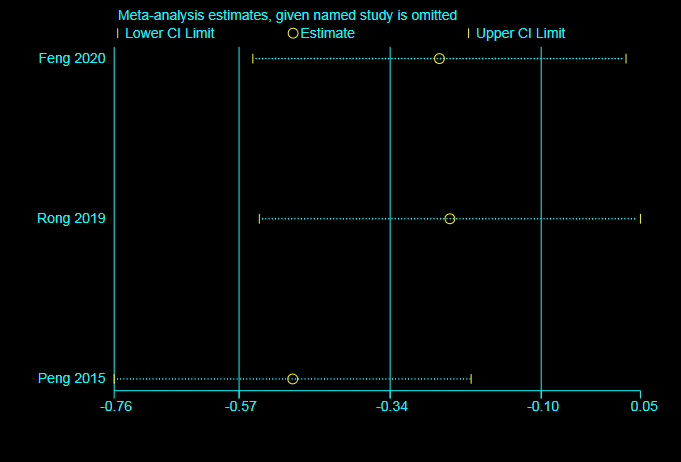


Supplementary figure 5: The sensitivity analysis of FINS.


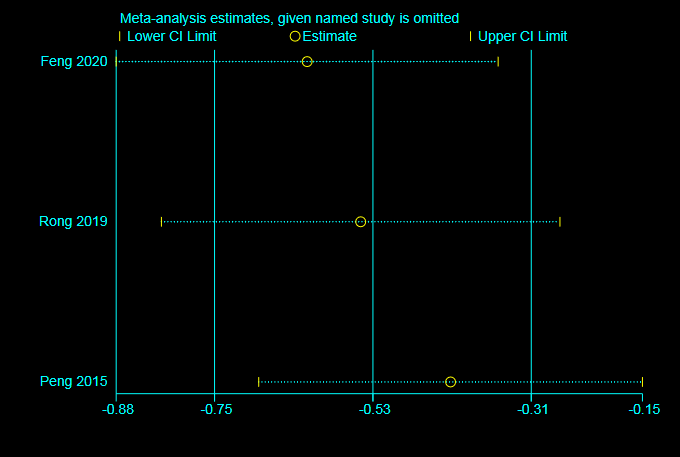


Supplementary figure 6: The sensitivity analysis of HOME-RI.
